# Step-by-Step Real-Time Electron Paramagnetic Resonance Monitored Protocol for Synthesizing a Nitroxide-Functionalized Periodic Mesoporous Organosilica

**DOI:** 10.1021/acs.chemmater.6c00530

**Published:** 2026-06-05

**Authors:** Tiago Morais, Satyaki Chatterjee, Mirtha A. O. Lourenço, Mariana Sardo, Ildefonso Marín-Montesinos, Snorri Th. Sigurdsson, Luís Mafra

**Affiliations:** † CICECO−Aveiro Institute of Materials, Department of Chemistry, 426216 University of Aveiro, 3810-193 Aveiro, Portugal; ‡ Department of Chemistry, University of Iceland, Science Institute, Dunhaga 3, 107 Reykjavik, Iceland

## Abstract

This protocol describes a step-by-step approach for the synthesis of a periodic mesoporous organosilica (PMO) with wall-embedded nitroxide monoradicals. It starts with the synthesis of an isoindoline-based nitroxide monoradical and its silylation—a critical requirement for the condensation with the PMO’s bisilylated building block. We also provide detailed instructions on how to synthesize the phenylene (Ph)-PMO’s bisilylated precursor, 1,4-bis­(triethoxysilyl)­benzene (BTEB), and subsequent preparation of the Ph-PMO with in situ incorporation of the silylated nitroxide monoradical. The incorporation of the nitroxide into the Ph-PMO was monitored by electron paramagnetic resonance (EPR) spectroscopy, which clearly revealed the decrease in free radical concentration in solution as the condensation progressed. A comparison of the radical-containing Ph-PMO to a reference Ph-PMO, prepared from the same organic precursor in the absence of the radical, by powder X-ray diffraction (PXRD), N_2_ adsorption–desorption isotherms, thermal gravimetric analysis (TGA), and Fourier transform infrared with attenuated total reflectance (FTIR-ATR), showed that the radical did not induce structural changes to the PMO. All reactions and methodologies described in this protocol were performed at least in triplicate, demonstrating their reproducibility. Completion of the entire protocol takes 22 days (∼3 weeks).

## Graphical abstract

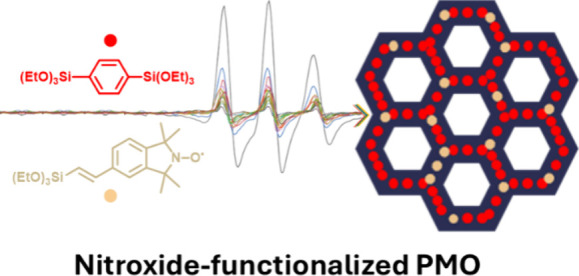

## Introduction

Embedding stable organic radicals into porous materials offers a powerful and generalizable approach to creating spin-active hybrid matrices. Such radical-functionalized materials have attracted increasing interest due to their potential utility in a wide range of applications, ranging from spectroscopic studies^1^ and investigation of materials’ surfaces,^2^ to environmental applications^3^ or even optoelectronic and spintronic applications.^4^

Several porous materials have been investigated as matrices for radical incorporation, including covalent organic frameworks (COF),^5^ metal–organic frameworks (MOF),^6^ carbon nanotubes^7^ and silica-based materials.^3^ Among silica-based materials, periodic mesoporous organosilicas (PMOs) represent a particularly promising but largely unexplored class for this purpose. PMOs are distinguished by the inherent and uniform distribution of organic groups within their silica framework,^8^ unlike conventional mesoporous silicas like SBA-15 or MCM-41, where functionalization typically relies on postsynthetic grafting.^9^ While PMOs share highly ordered, crystal-like mesostructures, with MOFs and COFs, they generally offer superior thermal and chemical stability,^10^ a significant advantage, whereas the limited stability of many MOFs and COFs remains a major research challenge.^10,11^ The primary limitation of PMOs, especially compared to conventional silicas, is the one-pot condensation of bis-silylated precursors, which often leads to low synthetic yields. Although this can be a drawback for large-scale applications, PMOs remain highly attractive for fundamental research. Their well-defined architecture makes them ideal model systems for studying structure–property relationships and exploring applications across diverse scientific fields.

Recent advancements have focused on incorporating radicals directly into silica-based materials via impregnation, grafting, or co-condensation techniques. The protocol presented here is a novel approach for the in situ incorporation of nitroxide monoradicals directly into the PMO walls. Additionally, the progress of these reactions in real time was monitored using electron paramagnetic resonance (EPR) spectroscopy. The advantages and limitations of this approach, compared to conventional strategies, are discussed in the following section.

## Discussion of Methodology

There are three main strategies for incorporating spin probes into solid adsorbents: impregnation (I), grafting (II), and co-condensation (III).

I. Impregnation is a physical process in which a radical is introduced into the pores of a material from a solution. In 2014, Lin et al.,^2^ studied the impregnation of three nitroxide radicals into a phenylene-bridged PMO. The results showed that the spin probes could be trapped within the micropores of the material, although the adsorption efficiency was highly dependent on the PMO’s synthesis conditions and was favored when the material had amorphous walls. While this technique preserves the original pore structure of the material, it may lead to inhomogeneous distribution and possible aggregation of the radicals.^12^

II. The grafting technique postsynthetically links radicals to the surface of the porous materials. For example, Gajan et al.^13^ synthesized silica-based materials with azide functional groups, which were subsequently converted to amines using the Staudinger reaction. Mono- and binitroxide radicals were subsequently coupled to these amines. According to the authors, a homogeneous radical distribution was achieved within the material by using this approach. Similar strategies were employed by two other groups. Gruning et al.^14^ studied the anchoring of two nitroxide radicals with different linkers and reported that this method enabled a uniform incorporation of the radicals while controlling their concentration. A comparable outcome was reported by Oliveira et al.,^1^ who grafted carboxy-proxyl nitroxide radicals into a SBA-15. While many authors highlight the ability of grafting to produce materials with a homogeneous distribution of radicals or other functional groups as an advantage, this claim is rarely supported by clear experimental evidence. Indeed, some studies have identified an inhomogeneous distribution of functional groups as a drawback of grafting.^15^ Nevertheless, an important advantage of grafting over impregnation lies in the covalent bonding of the functional groups to the material, which enhances their resistance to leaching during operation.^16^ A key limitation of grafting is potential pore blockage caused by accumulation of the grafted functional groups.^15^

III. Cocondensation is a synthetic process where two or more precursor molecules undergo simultaneous condensation, primarily used to prepare hybrid organic–inorganic silica-based materials.^17^ Two different strategies have been employed to incorporate radicals into silica-based materials via cocondensation. The first strategy employs organic spacers between face-to-face silylated radical precursors^18,19^ that protect the radical from reduction during the synthesis. Following formation of the silica network, removal of the spacers generates the radicals while enabling precise control over the distance between unpaired electrons embedded within the silica walls. The second strategy, demonstrated by Silverio et al.,^20^ involves a co-condensation of a tris-silylated nitroxide radical precursor with either tetraethyl orthosilicate (TEOS) alone or with TEOS and a bisilylated organic molecule. This approach produced hybrid silica-based materials with nitroxide radicals embedded within their walls. Notably, co-condensed radicals exhibited superior stability, compared to grafted counterparts, when exposed to ascorbic acid, showing minimal decomposition at equivalent concentrations.^20^ An additional advantage of cocondensation is that the probability of pore blockage is generally lower than in grafting, although it is not negligible. Putz et al.^21^ studied the functionalization of mesoporous silica with aminopropyl groups using both co-condensation and postgrafting approaches. For the postgrafted samples, a significant decrease in surface area and pore volume was observed, which is typically indicative of pore blockage. However, potential drawbacks of co-condensation include structural disruptions or heterogeneous distribution of functional groups within the silica framework.^15^

The main differences between impregnation, grafting, and co-condensation, as well as the advantages and disadvantages of each method, are summarized in Figure 1.

**Figure 1:**
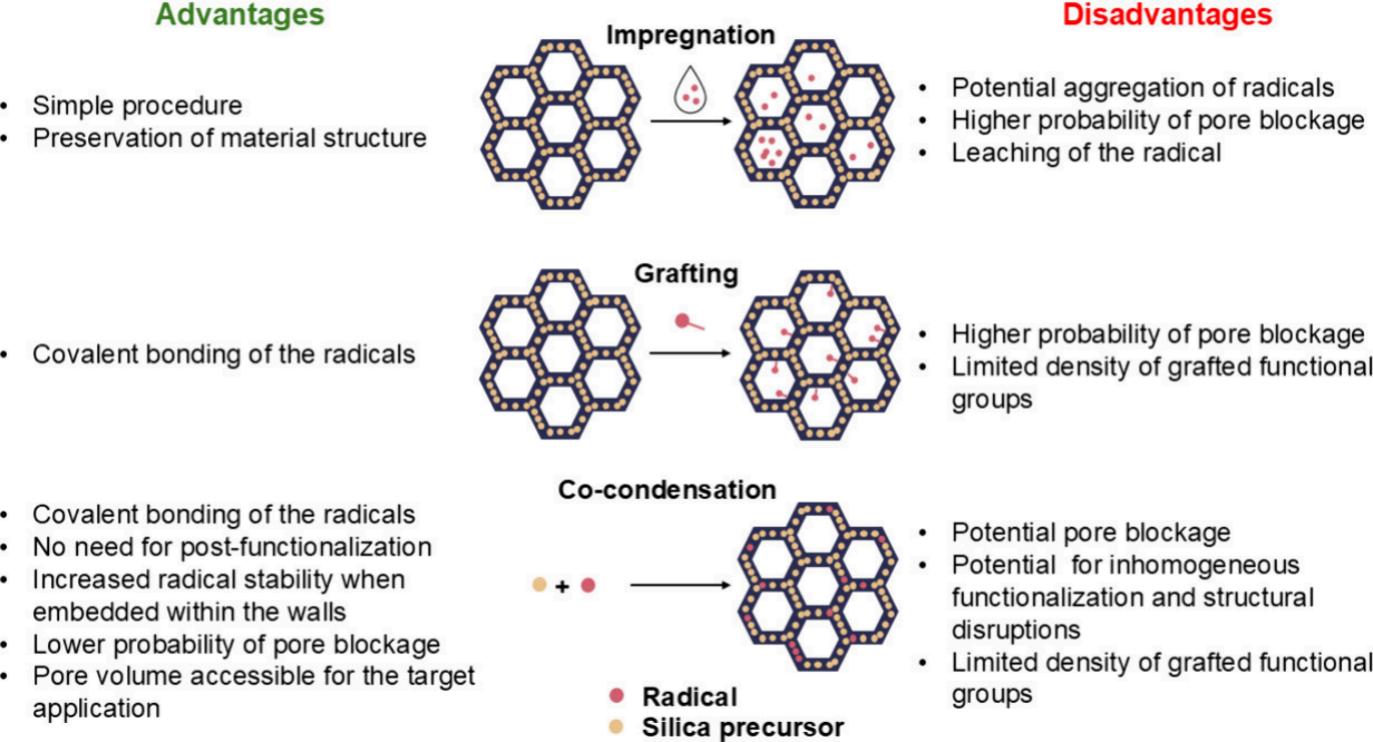
Comparison between the three methodologies for incorporating radicals into solid adsorbents: impregnation, grafting and cocondensation.

Of the aforementioned studies, only one reports the use of PMOs, specifically through radical impregnation into the pores.^2^ In contrast, our work demonstrates the in situ incorporation of nitroxide radicals directly within the PMO framework walls via co-condensation, while establishing PMOs as a versatile model system for diverse applications. In contrast to what is, to the best of our knowledge, the only existing report on radical incorporation into PMOs, which relied on impregnation, our approach not only prevents the leaching of the radicals from the pores but also decreases the probability of pore blockage, ensuring that the pore volume remains accessible for guest molecules. Although two initial strategies/attempts proved to be unsuccessful (described below), they provided valuable insights that guided our methodological refinements and ultimately enabled a successful implementation. The optimized protocols resulting from this development process are comprehensively detailed in the Procedure section.

### Protocol Development

Our initial approach involved the incorporation of the 4-hydroxy derivative of (2,2,6,6-tetramethylpiperidin-1-yl)­oxyl (TEMPOL), a nitroxide monoradical, into a silica-based material under acidic conditions, following an adapted protocol from the literature^20^ (Figure 2).

**Figure 2:**
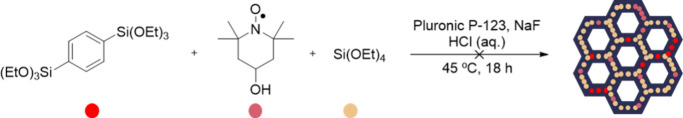
Schematic representation of the initial in situ incorporation of monoradicals under acidic conditions. The reaction did not yield the desired product, as indicated by the crossed-out reaction arrow.

TEMPOL was expected to undergo an alkoxy exchange reaction with TEOS,^22^ facilitating the formation of radical-incorporated silica material. The incorporation can be monitored in real time through EPR spectroscopy, where the broadening of the EPR line shape indicates restricted rotational mobility of the radical within the silica matrix.^23^ However, while monitoring the reaction, no EPR signal was detected from either the solution or the resulting solid materials. We hypothesized that the radical had been incorporated into the silica material but was reduced to an EPR-silent form. To test this hypothesis, the synthesized material was stirred in an aqueous solution of NaNO_2_ in an attempt to regenerate the radical,^24^ but unfortunately, no EPR signal was observed. These results suggest that TEMPOL is neither stable under acidic conditions nor is it effectively incorporated into the silica material in its active radical form. This behavior is consistent with the well-documented instability of nitroxide radicals under acidic conditions,^25,26^ and led us to investigate alkaline reaction conditions.

Cetyltrimethylammonium bromide (CTAB) was used as a pore structure-directing agent to adapt the synthesis for alkaline conditions (Figure 3).

**Figure 3:**
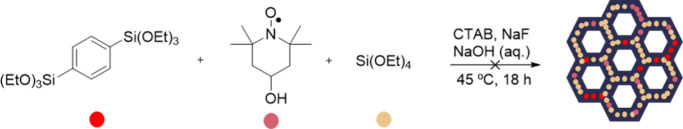
Schematic representation of the second attempt to incorporate a nitroxide monoradical in situ into PMO under alkaline conditions. The reaction did not yield the desired product, as indicated by the crossed-out reaction arrow.

Real-time EPR monitoring of the reaction mixture demonstrated that TEMPOL remained stable under the reaction conditions. The resulting solid material was subsequently washed, dried, and analyzed by EPR. However, no EPR signals were observed. This indicates that TEMPOL was neither covalently bound to the silica walls nor physically trapped within the pores of the material. To confirm this, TEMPOL was impregnated into a presynthesized phenylene-bridged PMO (Ph-PMO). The wet impregnated solid displayed the expected EPR signal for a nitroxide, but the material became EPR silent following filtration and drying, further confirming the lack of stable incorporation. This prompted us to attempt the incorporation of the radical during PMO synthesis under alkaline conditions, utilizing a presilylated radical precursor.

To preserve the periodic structure of the PMO, we selected an isoindoline nitroxide monoradical and employed a palladium-catalyzed silylation protocol adapted from the literature^27^ (Figure 4).

**Figure 4:**
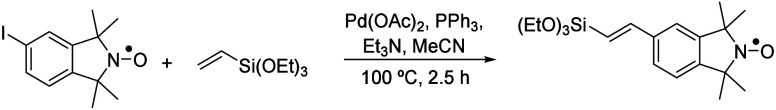
Scheme of silylated isoindoline nitroxide monoradical.

The success of this reaction was confirmed through EPR and high-resolution mass spectrometry (HRMS) analyses (results detailed in the Procedure section (compound **9**)). This reaction allows the preparation of a suitably derivatized radical precursor for subsequent co-condensation reactions.

The silylated monoradical was used to synthesize a silica-based material containing wall-embedded radicals by adapting a protocol from the literature^20^ (Figure 5).

**Figure 5:**
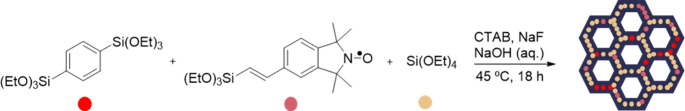
Schematic illustration of the in situ incorporation of monoradicals into a porous silica-based material. The reaction yielded the desired product.

The incorporation of the radical into the walls of a silica-based material was successful, serving as a proof of concept. The stability and progress of radical incorporation during the material synthesis were monitored in real time by EPR spectroscopy (Figure 6). The initial spectrum (Figure 6A) shows the characteristic three-line isotropic triplet of nitroxide radicals in a high-mobility environment, where fast rotational tumbling averages out the anisotropic hyperfine interactions. As co-condensation reaction progressed, these sharp peaks began to broaden, signaling a transition from a bulk solution environment to a constrained microenvironment within the forming silica matrix.^28^ This broadening was clearly visible after 60 min in Figure 6B (red circles), where the restricted rotational mobility of the radical within the rigid silica matrix resulted in an anisotropic spectral component. Simultaneously, the sharp peaks (red asterisks) represent the fraction of the radical that still remains as a free species in the solution. To confirm the presence of these two distinct environments, methanol (MeOH) was added to an aliquot of the reaction mixture (cf., Figure 6B) to precipitate the solid. The EPR spectrum of the decanted solvent (Figure 6C) shows the signal from the free radical isolated in solution. No significant changes were seen after 100 min (Figure 6D) or 18 h (Figure 6E), indicating that the reaction had reached completion, and a portion of the radical remained unincorporated. Following filtration and removal of the free-radical solution, the EPR spectrum of the solid material confirmed successful incorporation. Upon drying, the spectrum (Figure 6F) became significantly broader and more asymmetric. This was expected, since the radicals have been immobilized in the solid matrix, with the EPR signal representing a sum of all the possible orientations of the molecules with the magnetic field.^28^ The surfactant was subsequently extracted from the pores, and the EPR spectra of the resulting material were recorded in both the wet (Figure 6G) and dry (Figure 6H) states. The increased resolution and narrower line widths in the wet material indicate that the presence of solvent provides a less rigid local environment, allowing for a higher degree of rotational freedom compared to the fully dry state.^29^ These results demonstrate that EPR is an ideal tool for correlating spectral line shapes with the local radical environment, effectively monitoring both the successful incorporation and the degree of mobility within the silica-based matrix. Thus, we proceed to optimize and scale up the preparation, as well as characterizing the PMO material containing the wall-embedded monoradicals.

**Figure 6:**
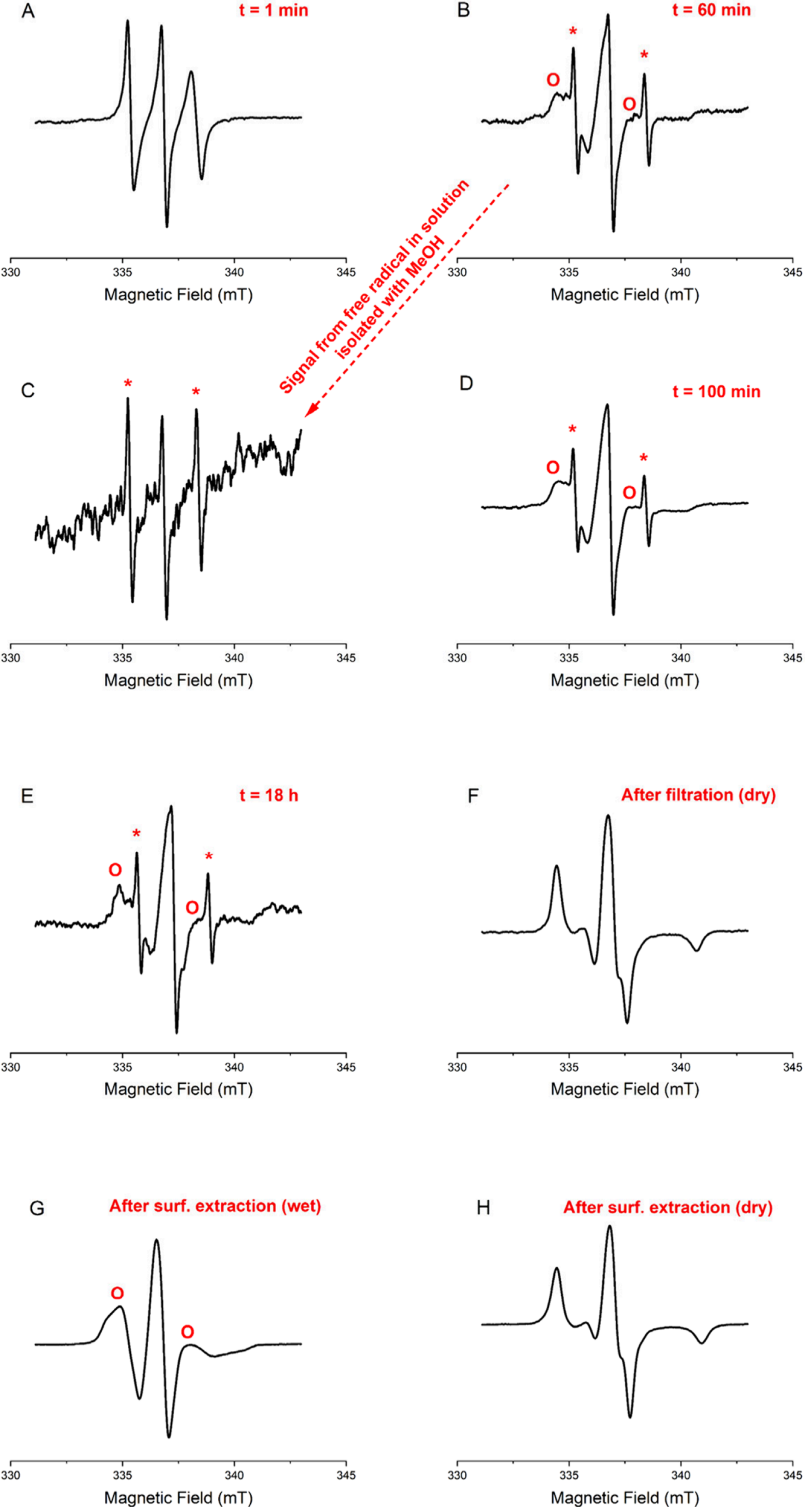
Real-time EPR monitoring of monoradical incorporation into the silica-based matrix. (A) Initial isotropic triplet of the free radical in solution (high rotational mobility). (B–E) Evolution of a composite spectrum showing the coexistence of the free radical (red asterisks) and the matrix-embedded radical (red circles); the latter exhibits significant line-broadening due to restricted rotational tumbling. (F) EPR spectrum of the dry solid after filtration. (**G–H**) Comparison of the material after surfactant extraction in wet vs dry states, illustrating the effect of solvent on the local radical mobility and the resulting transition from a partially resolved to a fully immobilized anisotropic powder pattern.

For the synthesis of radical co-condensed PMO, we adapted a protocol developed by Bion et al.^30^ (Figure 7) and successfully incorporated the nitroxide monoradical into the pore walls of the Ph-PMO. The yield from the first synthesis was insufficient for a full characterization of the material. Therefore, two more syntheses were performed, which not only provided enough material for further characterization but also confirmed the reproducibility of the method. A comparative analysis (PXRD, N_2_ adsorption–desorption isotherms, TGA and FTIR-ATR) between the reference Ph-PMO (without radical) and the radical-containing Ph-PMO (Ph-PMO_r) confirmed that the incorporation of the radical did not significantly affect either the structural or textural properties of the material (results in detail in the Material Characterization subsection). The real-time EPR monitoring of the synthesis showed consistent and reproducible results, further validating this methodology. The detailed results from various characterization techniques, along with the analysis and interpretation of the EPR data, are presented in the EPR Reaction Monitoring subsection. This iterative approach allowed us to advance from a proof-of-concept to a reproducible protocol for the in situ incorporation of nitroxide radicals into the walls of Ph-PMO.

**Figure 7:**
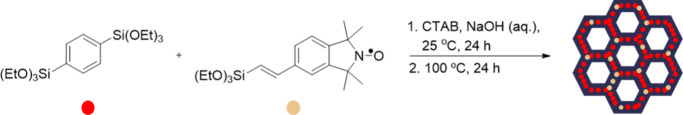
Schematic illustration of the in situ incorporation of monoradicals into a Ph-PMO.

The detailed procedure for this protocol is presented below.

## Procedure

The protocol has three main steps that provide flexibility, enabling users to adapt the procedure according to their specific needs. Users may choose to follow the complete protocol, starting with the synthesis of the silylated monoradical (Step 1), followed by its incorporation into the Ph-PMO material via co-condensation (Step 2). Simultaneously with Step 2, the optional monitoring of radical incorporation and stability using EPR spectroscopy can be performed (Step 3). Alternatively, users who already have a silylated monoradical can skip Step 1 and proceed directly to Step 2 for material synthesis – however, some adaptations may be needed. In some cases, users may opt to synthesize the radical in Step 1 but incorporate it into the material using different methods, thus using their own adapted Step 2. Monitoring with EPR spectroscopy in Step 3, while not essential for the success of the protocol, is recommended. It provides valuable insights into the incorporation of the radical, as well as the radical's stability and behaviour over time, which can guide the optimisation and application of the resulting material.

All reagents used in this protocol are described in the section Reagents from the Supporting Information.

### Step 1. Synthesis of the Silylated Monoradical

In this protocol, we describe in detail the synthesis of the silylated monoradical, which is a stable radical precursor for subsequent incorporation into the PMO framework (Figure 8). The target radical **9** (Figure 8) was synthesized according to methodologies previously reported in the literature.^31–33^ Starting with phthalic anhydride **1**, tertramethylisoindole **4** was obtained in three steps. Compound **4** was then subjected to acid-mediated nitration, followed by oxidation to give radical **6** in good yield. The nitro group was then reduced and iodinated to give compound **8**. Finally, the silylated isoindoline monoradical was synthesized by coupling vinyltriethoxysilane with radical **8**. To ensure successful reproduction, it is essential that the user carefully controls key parameters, such as catalyst loading and the moisture content of the solvents. The purity of all nonradical products should be verified by nuclear magnetic resonance (NMR) spectroscopy and the radicals by high-performance liquid chromatography (HPLC). Particular caution is required in the final step as the silyl groups are highly sensitive to moisture. Thus, freshly distilled acetonitrile (MeCN) must be used, and only the minimum amount of water should be added during quenching and workup (if required). All of the radical products should be analyzed by EPR spectroscopy. The schematic representation of the synthesis of compounds **2**–**9** is depicted in Figure 8.

**Figure 8:**
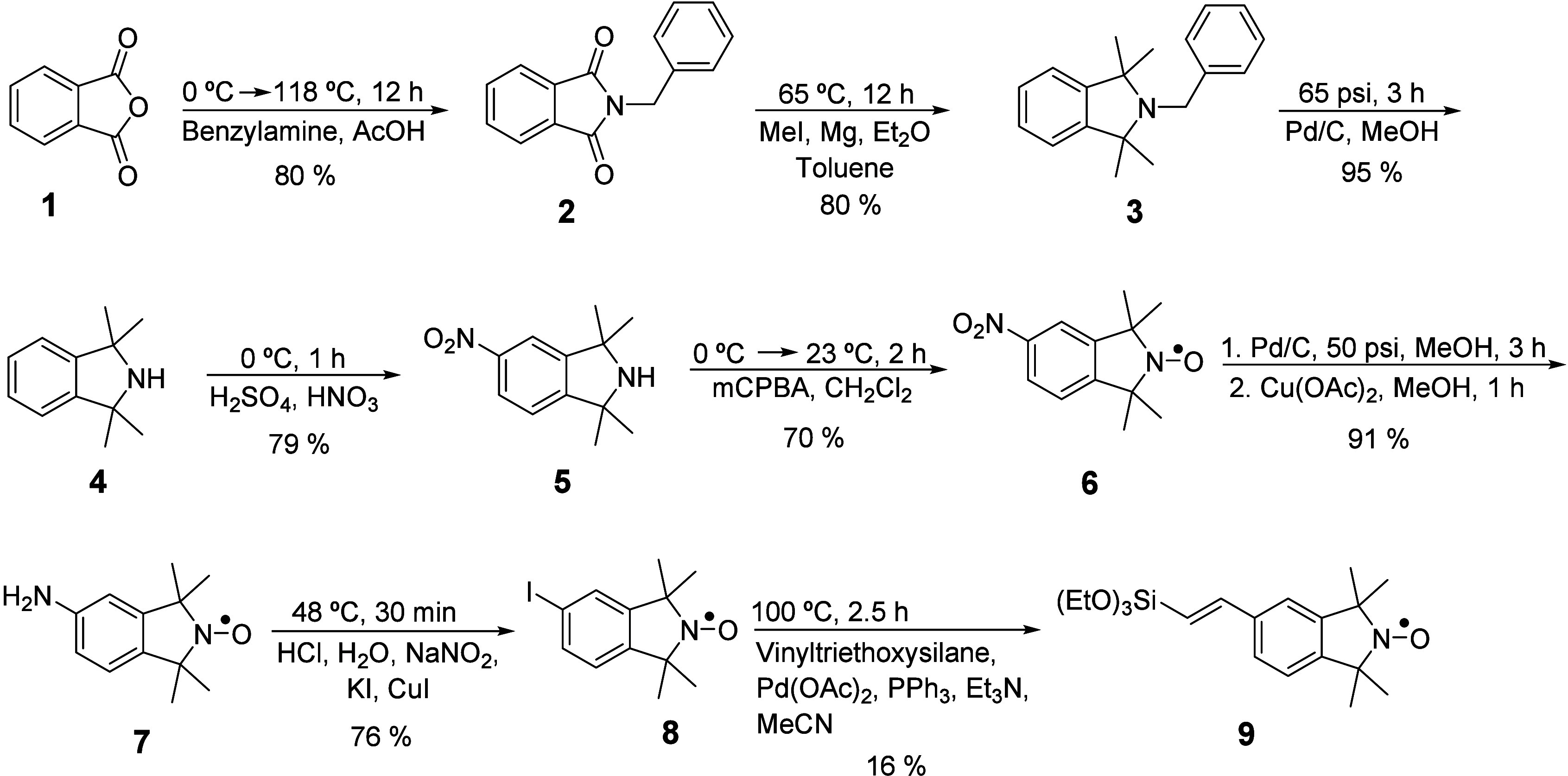
Multistep synthesis of the silylated isoindoline monoradical **9**.

The detailed procedure of each step is described below.

#### Synthesis of Compound **2**

(1) Phthalic anhydride (200 g, 1.35 mol) was added to a 1 L pear-shaped flask equipped with a magnetic stir bar. Glacial acetic acid was slowly added (500 mL).
(2) Cool the solution to 0 °C and add benzylamine (150 mL) dropwise to the stirring solution (note: without stirring of the reaction mixture, it is possible to see solid formation).
(3) Stir the reaction (including the partially formed solid) under reflux at 118 °C for 20 h.
(4) After 20 h, the reaction mixture was poured into crushed ice.
(5) Filter the formed product through a Buchner funnel and wash successively with water and ethanol (EtOH). Yield: 170 g (80%). ^1^H NMR (CDCl_3_, 400 MHz): δ. 7.84 (d, 2H,), 7.71 (d, 2H), 7.45 (d, 2H), 7.33 (m, 3H), 4.86 (s, 2H).

#### Synthesis of Compound **3**

(6) A 3-neck round-bottom flask was prepared, equipped with a condenser and a dropping funnel.
(7) Mg metal (25 g, 1.03 mol) was added.
(8) The glassware was dried along with the Mg metal by heating with a Bunsen burner under vacuum.
(9) The setup was flushed with argon.
(10) Diethyl ether (100 mL) and I_2_ (5 mol %) were added and stirred.
(11) A solution of iodomethane (100 mL, 1.61 mol) in diethyl ether (100 mL) was prepared and added to the dropping funnel.
(12) The iodomethane solution was added dropwise to the Mg-containing solution, at a rate that results in gentle reflux of the diethyl ether (note: This is an exothermic reaction, and thus, the addition needs to be slow).
(13) After addition, reflux the reaction mixture at 60–65 °C for 12 h (note: If the reaction is not done under sealed conditions, loss of diethyl ether is observed, which affects the yield of the compound **3**).
(14) Dilute the reaction with toluene (10 mL) and heat the reaction up to 110 °C to distill out the remaining diethyl ether.
(15) Compound **2** (25 g, 112 mmol) was dissolved in toluene (250 mL) (note: heat gently with a blow gun to help dissolution).
(16) Add the solution of compound **2** slowly to the reaction mixture over a period of 45 min at 90 °C.
(17) Heat the reaction mixture to 130 °C (under reflux) for 12 h.
(18) The reaction mixture was allowed to cool to room temperature.
(19) The reaction mixture was filtered through a Celite bed using a sintered funnel, and it was ensured that the solid on the bed did not dry (note: dry residue may ignite spontaneously). Petroleum ether was used for this filtration and for washing.
(20) After filtration, ethanol was added slowly to the solid residue over the Celite bed to quench the unreacted magnesium (note: bubbling effervescence observed).
(21) The solution was bubbled with air through the violet-colored filtrate (from step 15) overnight to quench excess magnesium salt.
(22) More petroleum ether (50 mL) was added the following day and filtered again through a small bed of Celite using a sintered funnel.
(23) The filtrate was concentrated to give a clear-yellow solid.
(24) Purify first by a short filter column (∼3 cm internal diameter × 20 cm length: ∼141 cm^3^ of volume) using basic aluminum oxide with 20 mL Pet. ether.
(25) Then purify by column chromatography using SiliaFlash Irregular Silica Gels F60 (∼5 cm internal diameter × 55 cm length: ∼1080 cm^3^ of volume) with 1% EtOAc/Pet. ether. to yield compound **3** as a white to pale yellow solid depending upon the extent of purity. Yield: 1.07 g (80%). TLC (Silica gel, pet. ether:EtOAc 30:1), R*f* (**6**) = 0.4. ^1^H NMR (CDCl_3_, 400 MHz): δ. 7.5 (d, 2H,), 7.34 (m, 5H), 7.21 (d, 2H), 4.10 (s, 2H), 1.45 (s, 12H).

#### Synthesis of Compound **4**

(26) Compound **3** (1 g, 3.8 mmol) was dissolved in MeOH (10 mL).
(27) Add Pd/C (100 mg, 90 μmol) to the previous solution (note: if performing this reaction on a large scale, cool the reaction mixture to 0 °C while adding Pd/C) and seal the reaction flask with a rubber stopper (septum).
(28) The reaction mixture was shaken under a positive pressure of hydrogen gas (at 65 psi) for 3 h, using a Parr hydrogenation apparatus (note: see equipment setup (Figure S1) in the Instrumentation section from the Supporting Information).
(29) The crude reaction mixture was diluted to a total volume of 20 mL with MeOH and filtered through Celite using a sintered funnel.
(30) Collect the filtrate and remove the solvent in *vacuo* at 40 °C using rotary evaporation, to yield compound **4** as a white solid. Yield: 627 mg (95%). ^1^H NMR (CDCl_3_, 400 MHz): δ. 7.34 (m, 2H), 7.15 (m, 2H), 1.52 (s, 12H).

#### Synthesis of Compound **5**

(31) Compound **4** (660 mg, 3.77 mmol) was dissolved in concentrated H_2_SO_4_ (6 mL) in an ice bath.
(32) Fuming of HNO_3_ (1.45 mL) was added in a stirring mixture (from (31)) over 10 min, and the reaction was allowed to stir for 1 h.
(33) The reaction mixture was poured into crushed ice and adjusted to pH 7.0 with 6 M NaOH (aqueous).
(34) The reaction mixture was extracted with EtOAc (2 × 100 mL). The phases were separated and the organic phase was washed with brine.
(35) Dry the combined organic phase over anhydrous Na_2_SO_4_. Stir for a few minutes and filter the solid Na_2_SO_4_. Evaporate the solvent with a rotary evaporator at 40 °C.
(36) Purify the crude by recrystallization using CH_3_CN to yield a light orange solid. Yield: 554 g (79%). ^1^H NMR (CDCl_3_, 400 MHz): δ. 8.13 (d, 1H), 7.96 (s, 1H), 7.24 (d, 1H), 1.5 (s, 6H), 1.49 (s, 6H).

Compound **5** can be stored safely at 25 °C for ∼3 years.

#### Synthesis of Compound **6**

(37) Compound **5** (1.5 g, 6.82 mol) was dissolved in CH_2_Cl_2_ (8 mL).
(38) Add *m*CPBA (1.64 g, >70 wt %, 10.23 mol) at 0 °C while stirring the reaction for 2 h at room temperature (∼20 °C).
(39) The reaction mixture was transferred to a separatory funnel and the organic layer was washed sequentially with NaHCO_3_ (1 M aq, 2 × 50 mL).
(40) Na_2_SO_4_ was added sufficiently to dry the organic solution (approximately 1 g), the flask was swirled occasionally for 5 min, and the salt was removed by filtration.
(41) Purify the crude over column using SiliaFlash Irregular Silica Gels F60 (∼5 cm internal diameter × 60 cm length: ∼1178 cm^3^ of volume) with an elution gradient from 4% to 6% EtOAc/Pet. ether to yield bright orange solid. Yield: 1.07 g (70%). TLC (Silica gel, CH_2_Cl_2_), R*f* (**6**) = 0.4. ESI-HRMS: *m*/*z* calculated [M + H]: 236.1161, found: 236.1167

#### Synthesis of Compound **7**

(42) Compound **6** (500 mg, 2.2 mmol) was dissolved in MeOH (10 mL).
(43) Add Pd/C (15 mg, 80 μmol) to the previous solution (note: if performing this reaction on a large scale, cool the reaction mixture to 0 °C while adding Pd/C) and stopper the reaction with a rubber septum.
(44) The reaction mixture was shaken under a positive flow of hydrogen gas (at 50 psi) for 3 h, using a Parr hydrogenation apparatus (note: see equipment setup (Figure S1) in the Instrumentation section from the Supporting Information).
(45) The crude reaction mixture was diluted to a total volume of 20 mL using MeOH and filtered through Celite using a sintered funnel.
(46) Cu­(OAc)_2_ (50 mg, 10 mol %) was added to the reaction mixture (from 45) and stirred for 1 h.
(47) Remove MeOH under *vacuo* at 40 °C and dilute with EtOAc (50 mL).
(48) The reaction mixture was transferred to a separatory funnel and the organic layer was washed sequentially with water until the blue coloration of the aqueous layer was completely gone.
(49) Purify the crude over column using SiliaFlash Irregular Silica Gels F60 (∼3 cm internal diameter × 30 cm length: ∼212 cm^3^ of volume) with an elution gradient from 15% to 20% EtOAc/Pet. ether to yield a beige-coloured solid. Yield: 450 mg (91%). TLC (Silica gel, pet. ether:EtOAc 8:2), R*f* (**7**) = 0.3. ESI-HRMS: *m*/*z* calculated [M + Na]: 228.1239, found: 228.1235.

Compound **7** can be stored safely at 4 °C for ∼3 years.

#### Synthesis of Compound **8**

(50) A heterogeneous mixture was prepared by adding compound **7** (350 mg, 1.59 mmol) in distilled water (2.1 mL).
(51) Add HCl (1.4 mL, 37%, aqueous) and stir it at 0 °C.
(52) Separately, in a 50 mL beaker, prepare a solution of NaNO_2_ (169 mg, 2.45 mmol) in distilled water (9.2 mL) and add it dropwise to the previous mixture, over a period of 10 min (note: addition time is scale-dependent).
(53) The reaction mixture was allowed to stir for 30 min.
(54) Separately, in a 100 mL beaker, CuI (32 mg, 0.17 mmol) and KI (1.4 g, 8.43 mmol) were dissolved in distilled water (53 mL).
(55) Under vigorous stirring, the reaction mixture (from (52)) was added to the aqueous solution (from 54) over a period of 10 min (note: addition time is scale dependent). Allow the reaction mixture to stir for 5 min at 23 °C and then warm the reaction mixture to 48 °C in 5 min and kept stirring at this temperature for 30 min.
(56) A saturated solution of Na_2_S_2_O_3_ was added to quench the excess I_2_ byproduct (note: iodine quenching can be observed through a color change from golden yellow to colorless).
(57) The reaction mixture was extracted with EtOAc (2 × 50 mL). The phases were separated and the organic phase was washed with brine.
(58) Purify over a column using SiliaFlash Irregular Silica Gels F60 (∼3 cm internal diameter × 30 cm length: ∼ 212 cm^3^ of volume) with 3 to 5% EtOAc./Pet. ether to yield yellow crystalline solid. Yield: 410 mg (76%). TLC (Silica gel, pet. ether:EtOAc 20:1), R*f* (**8**) = 0.3. ESI-HRMS: *m*/*z* calculated [M + Na]: 339.0096, found: 339.0101.

Compound **8** can be stored safely at 25 °C for ∼3 years.

#### Synthesis of Compound **9**

(59) Dry all the glassware at 120 °C before using it to avoid the presence of moisture and cool under inert conditions (note: the triethoxyvinylsilane reagent is moisture sensitive and, in the presence of water, will hydrolyze its ethoxy groups (−OCH_2_CH_3_) forming silanol groups (Si–OH). This can result in polymerization due to the condensation reaction between silanols).
(60) Add Pd­(OAc)_2_ (10.65 mg, 47 μmol), PPh_3_ (12.51 mg, 47 μmol), AgNO_3_ (26.86 mg, 0.16 mmol), Et_3_N (66 μL, 474 μmol) and compound **8** (50 mg, 158 μmol) in a round-bottom-flask and seal the reaction with a rubber septum. Dissolve the reagents in acetonitrile (2 mL). The glassware should be flushed with a flow of an inert gas, such as nitrogen or argon for several minutes, using a venting needle in the rubber septum (note: the acetonitrile should be freshly distilled before use in this reaction to ensure its dryness, avoiding the hydrolysis of ethoxy groups or other unwanted side reactions).
(61) Stir the reaction at 100 °C, under sealed conditions, for 2.5 h.
(62) Dilute the reaction mixture with dry EtOAc (10 mL) (note: the ethyl acetate should be dried over 4 Å MS before use).
(63) Remove the solvent *in* *vacuo* at 35 °C in the rotary evaporator.
(64) Purification of the reaction can be performed in two different ways according to the scale of the reaction:
  (a) Smaller scale reaction (<50 mg of radical): the product was dissolved in dichloromethane and purified over preparative TLC with an elution gradient of 12% EtOAc/Pet. ether to yield a yellow oil. Yield: 16%. TLC (silica gel, pet. ether:EtOAc 8:2), *Rf*(**8**) = 0.2.
  (b) Bigger scale reaction (>50 mg of radical): purify over a column using SiliaFlash Irregular Silica Gels F60 (∼2 cm internal diameter × 20 cm length: ∼63 cm^3^ of volume) with 5–7% EtOAc./Pet. ether to yield a yellow oil. Yield: 12%. ESI-HRMS: *m*/*z* calculated [M + Na]: 401.1993, found: 401.1973.

Compound **9** can be stored safely at −20 °C for <2 months.

Figure 9 illustrates the expected result from the EPR measurement of compound **9**.

**Figure 9:**
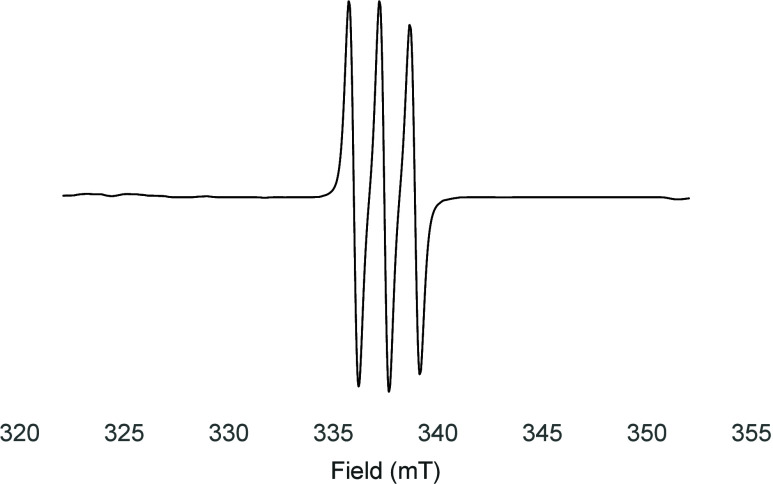
X-band CW-EPR spectrum of compound **9**, recorded in CH_2_Cl_2_ at 1 mM.

### Step 2. Synthesis of the Ph-PMO with Wall-Embedded Monoradical

The PMO was synthesized by cocondensing two precursors: 1,4-bis­(triethoxysilyl)­benzene (BTEB) and the silylated monoradical **9**. This reaction was conducted in an alkaline medium using CTAB as a structure-directing agent. For successful replication of this procedure, users must strictly follow all steps, as parameters such as pH, surfactant concentration, and temperature critically influence the structure of the final material. The silylated precursors require careful handling and must be stored under a dry, inert atmosphere (e.g., nitrogen or argon) to prevent exposure to moisture. Even traces of water present in ambient air can hydrolyze the ethoxy groups, forming silanols and promoting premature condensation. Special attention must also be given to the type of radical used. If a different radical from the one used in this protocol is used, its stability under the reaction conditions should be investigated. Following the synthesis, a thorough characterization of the PMO material is essential. Techniques such as powder X-ray diffraction (PXRD), N_2_ adsorption/desorption isotherms, thermogravimetric analysis (TGA), and EPR spectroscopy should be used to confirm successful incorporation of the radical and to evaluate the structural and textural properties of the resulting PMO material.

Detailed protocols for precursor synthesis and co-condensation are provided below.

#### Synthesis of Bisilylated Organic Precursor (BTEB)

(1) Dry all the glassware under vacuum at 120 °C before use to remove moisture (note: tetraethyl orthosilicate is sensitive to moisture, where ethoxy groups (−OCH_2_CH_3_) gets hydrolyzed to silanol groups (Si–OH); this leads to self-polymerization between silanols).
(2) Activate Mg in the oven at 120 °C for 4 h before use.
(3) A 3-neck round-bottom flask was assembled for the reaction, connected to a Schlenk line, and a vacuum was started; then, the system was purged with inert gas. The process was repeated for 3 cycles and the system was kept under a positive flow of inert gas.
(4) Add Mg (6 g, 247 mmol) in dry THF (60 mL) in the round-bottom flask, followed by TEOS (160 mL, 722 mmol) and a small amount of iodine crystals (2 or 3 crystals, a “spatula tip”). Allow it to stir for some time to homogenize.
(5) Separately, 1,4-dibromobenzene (18.4 g, 78 mmol) was dissolved in dry THF (50 mL) and transferred to a dropping funnel. The assembly was done in the 3-neck flask with the faucet turned off.
(6) Gradually heat to 50 °C and let it stir for a while.
(7) Add, dropwise, the solution of 1,4-dibromobenzene (from (5)) to the reaction mixture (from (4)) over 2 h under inert gas (note: this reaction is highly exothermic, so the addition must be done very slowly to prevent excessive heat buildup, boiling, or potential decomposition).
(8) Allow the reaction to stir for 30 min and gradually raise the temperature to reflux conditions (70 °C) and continue to reflux for 20 h.
(9) Gradually, the reaction was cooled to room temperature and the condenser and dropping funnel were removed under positive flow of inert gas (the most controlled way possible).
(10) THF was evaporated under vacuum, and dry *n*-hexane was added to precipitate salts and residues.
(11) Column filtration was performed under vacuum, followed by evaporation of hexane.
(12) Purify via distillation. We used a J-Kem’s Kugelrohr system:
  (a) 100 °C, 1.5 Torr: for solvent evaporation (1st collection flask)
  (b) 170 °C, 1.5 Torr: for other residues evaporation (1st collection flask)
  (c) 190–200 °C, 1.0 Torr: BTEB recovery (2nd collection flask)
  Yield: 10.85 g (34.5%). ^1^H NMR (500 MHz, CDCl_3_): δ 1.25 (t, 18 H), 3.86 (q, 12 H), 7.68 (s, 4 H).

BTEB can be stored safely under N_2_ at 25 °C for ∼2 years.

The step-by-step scheme for this synthesis (Figure S2) can be observed in the Synthesis section from the Supporting Information.

#### Synthesis of Reference Ph-PMO

(13) Dissolve CTAB surfactant (4.8 g, 13.2 mmol) in an aqueous solution of NaOH (131.6 mL, 0.35 M) at 50 °C under stirring. After dissolution, gradually decrease the temperature to ∼30 °C.
(14) Add, dropwise, BTEB (4.8 g, 12 mmol) to the reaction mixture (from 13) while stirring at room temperature. Solution addition rate: ∼0.5 mL/min.
(15) The reaction mixture was sonicated for 20 min at 120 W and then stirred vigorously (800 rpm) for 24 h at room temperature, using a cylindrical PTFE stirring bar (35 mm length, 4 mm diameter, or similar).
(16) Transfer the mixture (from (15)) to a 200 mL Teflon container and place inside a reactor for hydrothermal treatment at 100 °C for 24 h. Preheat the oven to 100 °C before placing the reactor.
(17) The mixture was allowed to cool, and then the solid was filtered using a sintered funnel with a porosity of 4. The solid was washed with warm distilled water.
(18) Collect the solid and dry in the oven at 70 °C for 24 h.
(19) Extract the surfactant with a solution of HCl (2.2 mL, 12 M) in ethanol (150 mL) under reflux (80 °C) for ∼20 h.
(20) The mixture was allowed to cool before proceeding with filtration using a glass funnel with a porosity of 4. The solid was washed with ethanol and rinsed with distilled water.
(21) Collect the solid and dry in the oven at 70 °C for 24 h to yield the Ph-PMO.

The step-by-step scheme for this synthesis (Figure S3) can be observed in the Synthesis section from the Supporting Information.

#### Synthesis of PMO with in Situ Incorporation of Radical

(22) Dissolve CTAB (1.2 g, 3.4 mmol) surfactant in an aqueous solution of NaOH (32.9 mL, 0.35 M) at 50 °C under stirring. After dissolution, gradually decrease the temperature to ∼30 °C.
(23) Separately, mix BTEB (1.2 g, 3 mmol) and compound **9** (6 mg, 15 μmol) (note: use sonication for ca. 1 min to facilitate dissolution).
(24) Add, dropwise, the reaction mixture (from (23)) in the surfactant solution (from (22)) while stirring at room temperature. Solution addition rate: ∼0.5 mL/min.
(25) The reaction mixture was sonicated for 20 min at 120 W and then stirred vigorously (800 rpm) for 24 h at room temperature, using a cylindrical PTFE stirring bar (35 mm length, 4 mm diameter, or similar).
(26) Transfer the mixture (from (25)) to a 50 mL Teflon container and place inside a reactor for hydrothermal treatment at 100 °C for 24 h. Preheat the oven to 100 °C before placing the reactor.
(27) The mixture was allowed to cool, and then the solid was filtered using a sintered funnel with a porosity of 4. The solid was washed with warm distilled water.
(28) Collect the solid and dry in the oven at 70 °C for 24 h.
(29) Extract the surfactant with a solution of pyridine (11.28 mL, 140 mmol) and HCl (2.2 mL, 12 M) in distilled water (11.28 mL), under reflux (80 °C) for ∼20 h.
(30) The solid was refiltered using a glass funnel with a porosity 4. The solid was washed with ethanol and rinsed with distilled water.
(31) Dry the solid in the oven at 70 °C for 24 h to yield the Ph-PMO_r.

Representative photographs of selected synthetic steps are provided in the Synthesis section of the Supporting Information.

### Step 3. EPR Monitoring of the Reaction

For users opting to monitor the reaction in real time via EPR spectroscopy, access to an EPR spectrometer throughout the PMO synthesis is essential. Ideally, both the synthesis and the EPR measurements should be performed in proximity to minimize delays between sampling and analysis. To ensure reliable and comparable results, the measurement parameters must remain consistent across all time points. Additionally, users must have access to a centrifuge to separate the solid materials from the supernatant. This ensures that the analysis is performed exclusively on the radicals dissolved in the liquid phase.

To monitor the in situ incorporation of the silylated radical into the walls of the Ph-PMO, aliquots are removed at different time points and placed in an Eppendorf for analysis. The first time this procedure is carried out, frequent time points are recommended. As an example, in this protocol the following time points were used:

(1) t_0_ (right after the dropwise addition of BTEB/radical)
(2) t = 20 min (after 20 min of ultrasound)
(3) t = 34 min
(4) t = 52 min
(5) t = 70 min
(6) t = 91 min
(7) t = 108 min
(8) t = 144 min
(9) t = 163 min
(10) t = 182 min
(11) t = 211 min
(12) t = 241 min
(13) t = 1440 min (24 h) (after 24 h of stirring at room temperature)
(14) t = 2880 min (48 h) (after 24 h of hydrothermal treatment)

To measure the free radical in solution, we centrifuged the samples until the solid part separated. The liquid part of the sample was transferred to an EPR tube and measured in the EPR spectrometer. The parameters used were the following: 3480 G center field, 600 G sweep width, and 3480 G static field. Frequency of 9.771 GHz and power of 2 mW. Receiver gain of 2.00 × 10^3^, modulation frequency of 100 kHz, modulation amplitude of 16.35 G, time constant of 81.92 ms, conversion time of 40.96 ms, and sweep time of 41.94 s. Resolution in X was 1024 and 4 X-scans were used.

## Troubleshooting and Safety

The purpose of this section is to describe potential troubles that may occur and provide some hints to overcome them. Below you will find a troubleshooting table (Table 1) with some specific problems that may occur during the protocol, the possible reasons, and solutions.

**Table 1:** Troubleshooting

Step	Problem	Possible reason	Solution
**1** (16)	Reaction was boiling vigorously, and solution became dark.	Solvent was not dry.	Keep the reaction going and purify the product carefully later.
		Adding was too fast.	
**1** (25)	Product not formed or the yield is very low.	Magnesium was not activated before.	Activate the magnesium prior to reaction.
		Solvent was not dry.	Use dried solvent.
**1** (30)	Product formed but starting material not consumed.	Pd/C might not be in active form anymore.	Activate the Pd/C, add more to the reaction mixture and continue the reaction for 1 h more.
**1** (49)	Product formed but starting material not consumed.	Pd/C might not be in active form anymore.	Activate the Pd/C, add more to the reaction mixture and continue the reaction for 1 h more.
**1** (58)	Byproduct formation via the Ullmann Reaction.	Slow addition.	Addition needs to be rapid, while stirring at <200 rpm.
**2** (7)	Reaction was boiling vigorously, and the reaction became dark.	Adding was too fast.	Dispose of the waste and restart the reaction.
**2** (12)	Product not pure.	Mixture with residues with lower b.p.	Slowly increase the temperature during distillation and check if anything is being evaporated.
		NMR tubes are dirty.	Use new or clean tubes.
	Product not formed.	Reagents are contaminated or may contain water.	Check it with NMR.
**2** (17)	Small amount of material.	pH is too low.	Add some drops of HCl to induce precipitation of material (note: cannot do it for the radical-containing material as it can reduce it)

There are also some general factors that must be considered when following this protocol.

### Moisture

The presence of moisture, even in trace amounts, may be sufficient to interfere with the reaction, as shown in Table 1. Particular care should be taken when working with silylated compounds, as moisture promotes hydrolysis and polymerization. An inert atmosphere should be used during these reaction steps, and all of the solvents must be thoroughly dried.

### Radical Stability

When working with radicals, a critical aspect is the stability. As shown in the Protocol Development section, the nitroxide radical used here was not stable under the acidic conditions tested. Careful control of the pH is, therefore, required to avoid such harsh conditions. A useful practice is to routinely monitor radical stability by using EPR.

## Characterization and Validation

### Material Characterization

To assess the impact of the radical functionalization on the PMO, a reference Ph-PMO was also prepared, and the characterization results of both materials were compared. All materials synthesiz ed using this protocol should be characterized by the standard techniques. The instruments used in this work are described in the Instrumentation section of the Supporting Information. PXRD was used to evaluate the structural order of the materials, and Figure 10a shows the PXRD patterns of the reference PMO and the radical-functionalized Ph-PMO (Ph-PMO_r). Both diffractogram overlap and display an intense low-angle reflection and two much less intense peaks, corresponding, to the (100), (110) and (200) reflections characteristic of the two-dimensional hexagonal symmetry (*P*6*mm*) lattice. This pattern is typical in Ph-PMOs.^34^ Figure 10b shows the TGA curves for both materials. The first weight loss is attributed to desorption of water and other gases occurring below 100 °C. Above 550 °C, the thermal stability of both materials, shows a decomposition related to the degradation of the phenylene bridges present in the frameworks. Figure 10c shows the N_2_ adsorption–desorption isotherms at −196 °C, being exhibited characteristic type IV isotherms, and Figure 10d shows the narrow distribution of pore sizes, both typical of mesoporous (2–50 nm) materials.^35^ By combining the data from PXRD and N_2_ adsorption–desorption isotherms, it is possible to calculate some structural properties that are presented in Table 2. By analyzing it, it is observed that both materials present similar properties. The pore volume (V_P_) presents a slight decrease in the Ph-PMO_r, which can be explained by the introduction of the radical into the material. Figure 10e shows the FTIR-ATR spectra, being clear the overlap of the bands of both materials, meaning that no significant differences are seen by this technique also. Figure 10f shows the EPR spectra of the Ph-PMO_r, exhibiting a typical pattern from a nitroxide radical with low rotational mobility.^36^ All the characterization data proves that the radical was successfully introduced into the Ph-PMO, and that the radical did not induce structural changes in the PMO. The Ph-PMO_r was synthesized two times to assess reproducibility, confirmed by the similar characterization results (Figure S4 and Table S1, Reproducibility section from the Supporting Information).

**Table 2:** Structural Properties of Ph-PMO and its Monoradical-Containing Analogue (Ph-PMO_r), Derived from PXRD and N_2_ Adsorption-Desorption Isotherms Measured at −196 °C

Sample	*d*_100_ (nm)^a^	*a* (nm)^b^	*S*_BET_ (m^2^/g)^c^	*V*_P_ (cm^3^/g)^d^	*d*_P_ (nm)^e^	*b* (nm)^f^
**Ph-PMO**	4.12	4.76	786	0.56	2.22	2.54
**Ph-PMO_r**	4.07	4.70	770	0.48	2.20	2.50

a *d*_100_ is the interplanar spacing obtained from PXRD.

b Unit cell parameter (*a*) calculated as (2*d*_100_/√3).

c The specific surface area (*S*_BET_) was calculated by the BET method.

d The total pore volume (*V*_p_) was obtained from the adsorption data.

e Pore diameter (*d*_p_) obtained from the BJH method.

f Pore wall thickness (*b*) calculated as (*a* – *d*_P_), where the first term is the unit cell parameter.

**Figure 10:**
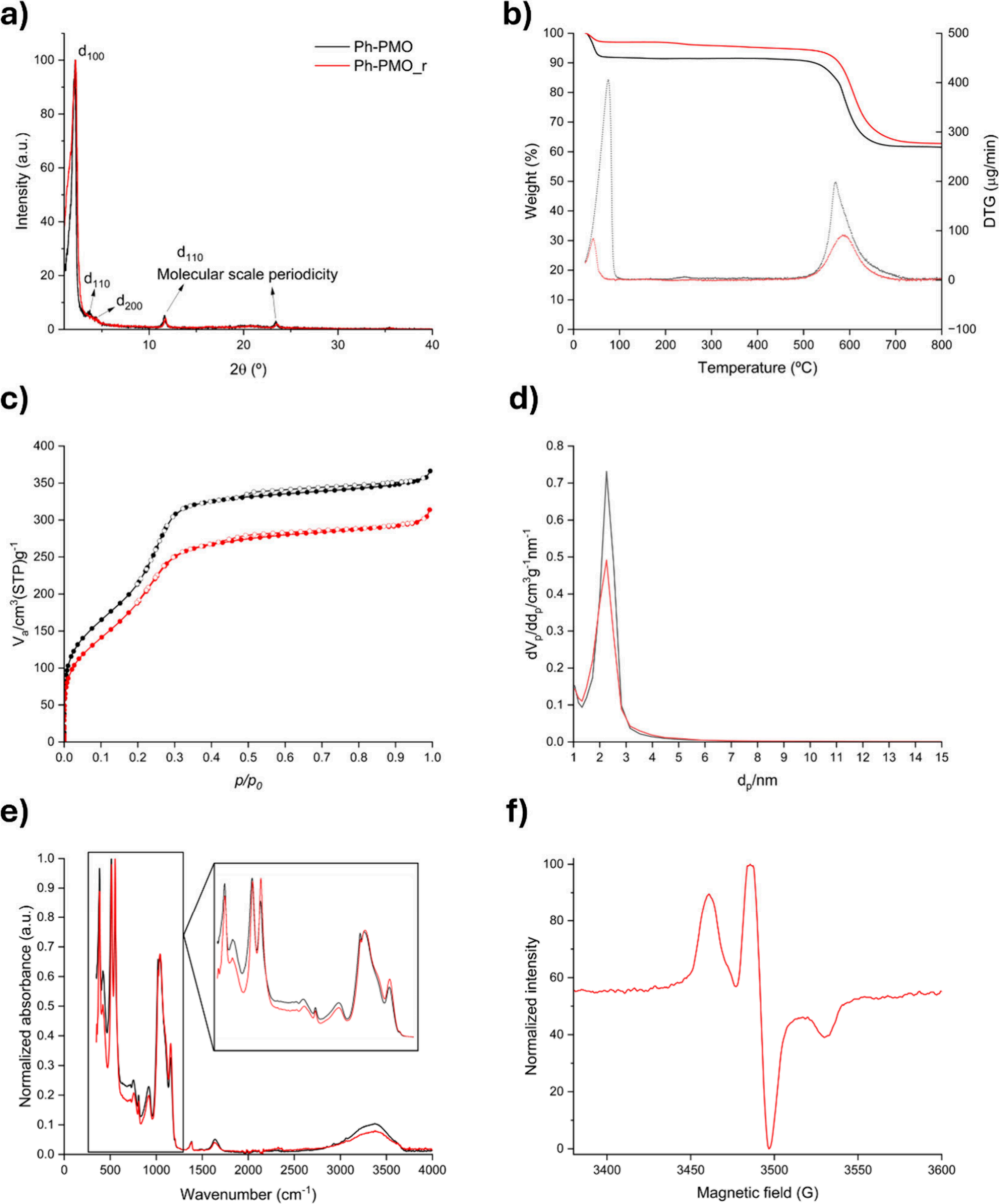
Characterization of the reference Ph-PMO (black) and Ph-PMO with incorporated monoradical (red): (a) PXRD, (b) TGA, (c) N_2_ adsorption–desorption isotherms, (d) pore size distribution curves, (e) FTIR-ATR, (f) EPR.

### EPR Reaction Monitoring

The kinetics of the radical-containing Ph-PMO synthesis were monitored using EPR spectroscopy by tracking the concentration of the monoradical remaining in the liquid phase over time. Figure 11 (left) displays the EPR spectra of aliquots taken from the reaction mixture at various intervals. A significant decrease in the signal intensity is observed as the reaction progresses, which directly correlates with the incorporation of the radical species from the isotropic liquid phase into the growing PMO framework. A plot of the integrated EPR peak area versus reaction time (Figure 11 (right)) reveals that the rate of incorporation is highest during the initial stages of the synthesis. The loss of signal in the liquid phase serves as proxy for the successful portioning of the radical into the rigid silica-based walls, which exhibits the severe broadening characteristic of immobilized species (as seen in Figure 6). The reproducibility of this kinetic study was validated in a repeated experiment (Figure S5, Supporting Information).

**Figure 11:**
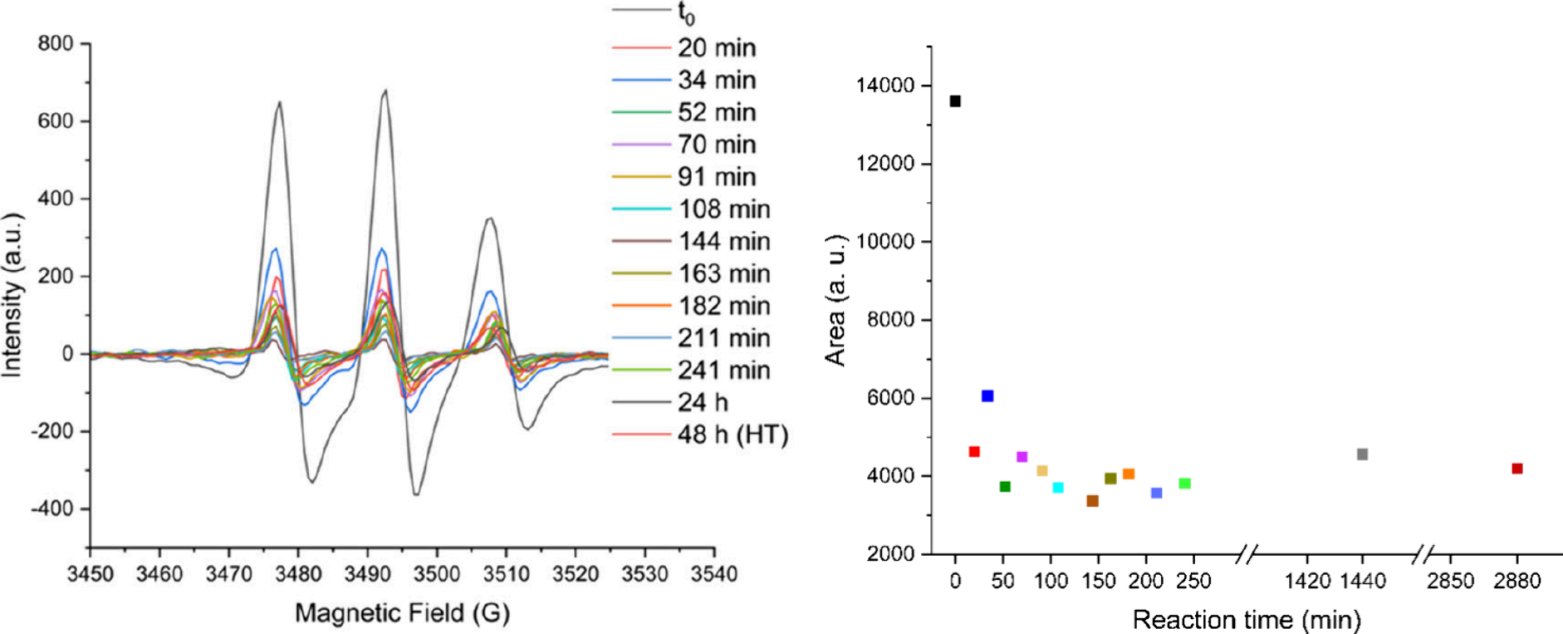
Kinetic monitoring of the Ph-PMO synthesis via liquid-phase EPR spectroscopy. (left) Time-resolved EPR spectra showing the decay of the isotropic nitroxide signal as the radicals are incorporated into the solid matrix. (right) Integration of the EPR peak areas as a function of reaction time, illustrating the rapid initial rate of radical entrapment and the eventual stabilization of the residual radical concentration in solution.

## Protocol Limitations

### Timing

The entire protocol, from radical synthesis to its incorporation into Ph-PMO and subsequent EPR monitoring, takes approximately 22 days. While this duration is acceptable for fundamental research applications, it may represent a limitation for large-scale or industrial implementations. Although reducing the overall processing time was not the primary focus of this study, several steps could potentially be accelerated without compromising the final product. For instance, the PMO synthesis, which currently relies on conventional thermal heating, could be significantly shortened using microwave-assisted methods, which have been reported to reduce reaction times from days to hours in similar organosilica systems by enabling rapid and homogeneous heating.^37^

### Yield

The syntheses of both the silylated nitroxide and the PMO are associated with relatively low yields. This may limit the scalability of radical-functionalized PMOs and related mesoporous silica materials for large-scale applications.

### Pore Blockage

Although cocondensation minimizes pore blockage compared to grafting or impregnation, high concentrations of the radical precursor, if not carefully controlled, may lead to localized structural disruptions or a reduction in the long-range mesoscopic order of the PMO framework.

## Conclusions

In this work, we have described a comprehensive, step-by-step protocol for the in situ incorporation of nitroxide monoradicals into the framework walls of a Ph-PMO, which can be extended to other silica architectures that rely on similar hydrolysis and condensation mechanisms. The protocol here presented is structured into three steps: (i) the multistep synthesis of a silylated isoindoline nitroxide radical, (ii) the process to form the radical-functionalized PMO, and (iii) the real-time monitoring of the reaction via EPR spectroscopy. By detailing the optimized parameters and highlighting the lessons learned from initial unsuccessful attempts, this protocol ensures a high reproducibility for the materials chemistry community. The described EPR monitoring procedure not only validates the successful covalent embedding of the radicals but also provides a versatile analytical framework that can be adapted to the study of the synthesis of other spin-active hybrid materials. We anticipate that this protocol will lower the barrier for the development of radical-functionalized PMOs in diverse fields, including catalysis, environmental remediation, and the study of surface-guest interactions.

## Supplementary Material



## Notes

Reagents, instrumentation, photos from synthesis, reproducibility data, and safety measures (PDF)
