# Supplementary material for: Step-by-Step Real-Time Electron Paramagnetic Resonance Monitored Protocol for Synthesizing a Nitroxide-Functionalized Periodic Mesoporous Organosilica
Source: Chem Mater. 2026 Jun 5;38(12):5864–77. doi: 10.1021/acs.chemmater.6c00530 (PMC13296274; doi:10.1021/acs.chemmater.6c00530)
Supplement: Supplementary file 1 [file cm6c00530_si_001.pdf]

A step-by-step real-time Electron Paramagnetic Resonance monitored protocol for synthesizing a nitroxide-functionalized periodic mesoporous organosilica

Tiago Morais[1,2], Satyaki Chatterjee[2], Mirtha A. O. Lourenço[1], Mariana Sardo[1], Ildefonso Marín-Montesinos[1], Snorri Th. Sigurdsson[2], and Luís Mafra[1]

[1] CICECO – Aveiro Institute of Materials, Department of Chemistry, University of Aveiro, 3810-193, Aveiro, Portugal

[2] Department of Chemistry, University of Iceland, Science Institute, Dunhaga 3, 107 Reykjavik, Iceland

Reagents

1,4-Dibromobenzene (Sigma-Aldrich, 98 %), acetic acid (AcOH) (Fluka, 99.8-100.5 %), acetonitrile (ChemeGenes, wash grade), benzylamine (Sigma-Aldrich, 99 %), copper (I) iodide (CuI) (Sigma-Aldrich, 99.9 %), copper (II) acetate ($Cu(OAc)_2$) (Sigma-Aldrich, 98 %), dichloromethane (CH_2Cl_2) (Honeywell, ≥ 99.8 %), diethyl ether (Et_2O) (Sigma-Aldrich, 99.8 %), ethyl acetate (EtOAc) (Sigma-Aldrich, 99.7 %), hexadecyltrimethylammonium bromide (CTAB) (Sigma-Aldrich, for molecular biology, ≥ 99 %), hydrochloric acid (HCl) (Honeywell, 37 %), iodine bisublimated (I_2) (Carlo Erba, p.a.), iodomethane (MeI) (Sigma-Aldrich, 99 %), magnesium (Sigma-Aldrich, for Grignard reactions, ≥ 99 %), meta-Chloroperoxybenzoic acid (*m*CPBA) (Sigma-Aldrich, 77 %), methanol (MeOH) (Honeywell, ≥ 99 %), n-Hexane (Carlo Erba, 99 %), nitric acid (HNO_3) (Sigma-Aldrich, ≥ 99.5 %), palladium (II) acetate ($Pd(OAc)_2$) (Sigma-Aldrich, 98 %), palladium on carbon (Pd/C) (Sigma-Aldrich, 37-40 %), petroleum ether (Pet. Ether) (Sigma-Aldrich, 95 %), phthalic anhydride (Sigma-Aldrich, 99 %), potassium iodide (KI) (Acros, 99 %), silver nitrate ($AgNO_3$) (Merck, GR grade), sodium bicarbonate ($NaHCO_3$) (Skeljungur, food grade), sodium hydroxide (NaOH) (Honeywell, pellets (anhydrous), ≥ 98 %), sodium nitrite ($NaNO_2$) (Sigma-Aldrich, 97 %), sulfuric acid (H_2SO_4) (Sigma-Aldrich, 95-98 %), tetraethyl orthosilicate (TEOS) (Sigma-Aldrich, reagent grade, 98 %), tetrahydrofuran (THF) (Carlo Erba, anhydrous stabilized with BHT), toluene (Honeywell, ≥ 99.7 %), triethylamine (Et_3N) (Sigma-Aldrich, ≥ 99 %), triphenylphosphine (PPh3) (Sigma-Aldrich, 99 %) were used as received without further purification.

Instrumentation

Powder X-ray diffraction (PXRD) patterns were collected in transmission mode on a PANalytical Empyrean diffractometer over a 2θ range of 0.5–40°, with a step size of 0.026°, 1504 data points, and a counting time of 96.39 s per step.

Thermogravimetric analyses (TGA) were carried out in air using a Hitachi NEXTA STA 300 instrument over a temperature range of 25–800 °C, with a heating rate of 5 °C min^{-1}. High-resolution mass spectra (ESI-HRMS) were acquired on a Bruker micrOTOF-Q spectrometer.

Nitrogen adsorption–desorption isotherms were measured using a BELSORP MAX II HP instrument. Prior to analysis, samples were degassed at 120 °C under vacuum for 4 h using a heating ramp of 5 °C min^{-1}. Measurements were performed at −196 °C.

FTIR spectra were recorded on a Bruker Tensor 27 spectrometer equipped with an ATR Golden Gate accessory (SPECAC), using a resolution of 4 cm^{-1}, 256 scans, and a spectral range of 4000–400 cm^{-1}.

EPR measurements were performed using a Bruker EMX EPR spectrometer (University of Coimbra) and a Magnettech MiniScope MS200 EPR spectrometer (University of Iceland).

HPLC analyses were carried out on an Agilent 1200 Series analytical system equipped with a NUCLEODUR C18 Pyramid column (4.6 × 150 mm) and UV detection at λ = 254 nm, using isocratic 100% CH_3CN at a flow rate of 0.75 mL min^{-1} for 15 min.

Liquid-state NMR spectra were recorded on a Bruker Avance III HD 500 spectrometer (University of Aveiro) and a Bruker 400 UltraShield spectrometer (University of Iceland). Samples (~20 mg) were dissolved in ~0.5 mL of deuterated chloroform ($CDCl_3$), and spectra were acquired using 32 scans at 400 MHz.

Hydrogenation reactions were performed using a Parr hydrogenation apparatus (**Figure S1**). Samples were placed in a Pyrex bottle, which was flushed three times with H_2 gas before pressurization to the desired pressure (psi). The system was connected to a water aspirator for pressure release, and hydrogen pressure was monitored over 30 min to ensure stable reaction conditions. **Figure S3** describes all the setup for this method.

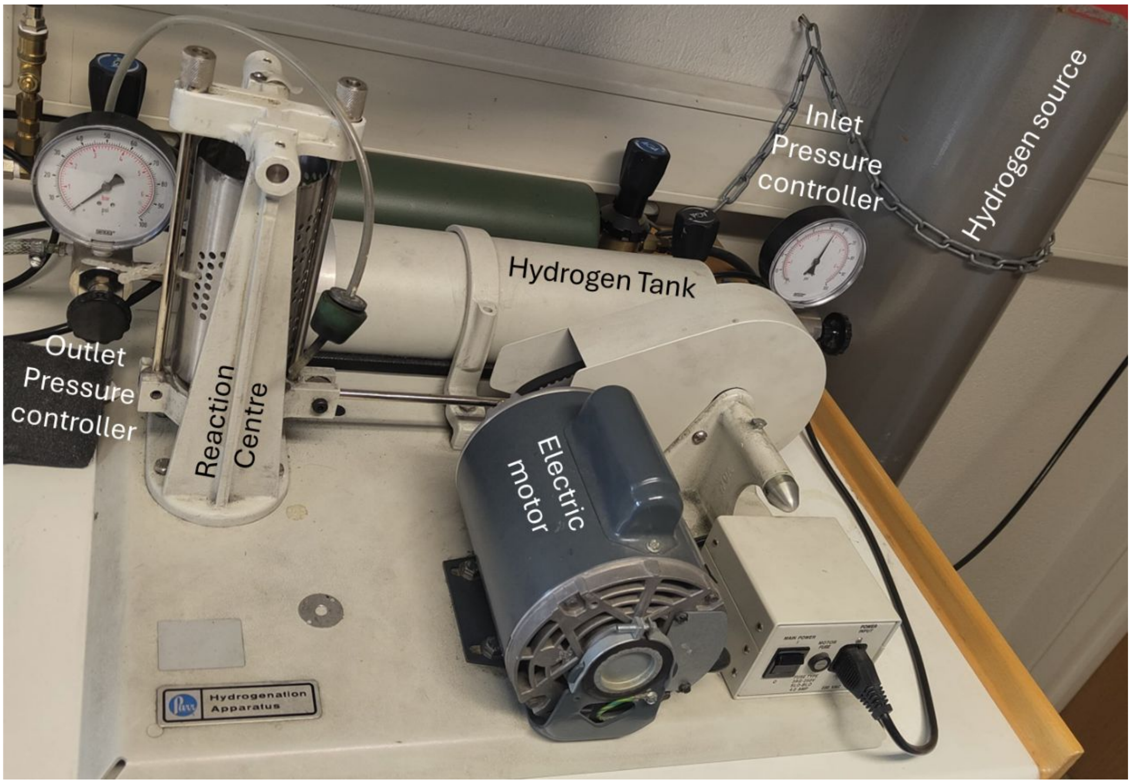

Figure S1. Parr hydrogenation apparatus.

Synthesis

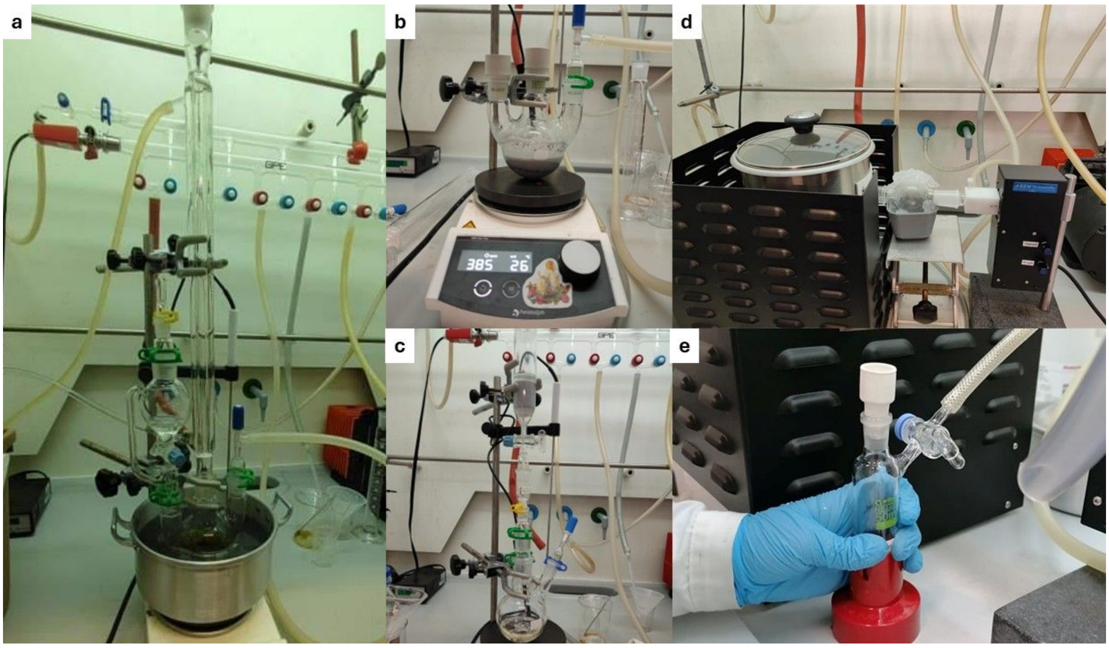

Figure S2. Step-by-step scheme for the synthesis of BTEB (precursor for Ph-PMO): a) Initial setup under inert atmosphere including 3-neck round-bottom flask, dropping funnel and condenser. b) THF evaporation under vacuum. c) Column filtration followed by evaporation of hexane. d) Distillation using a J-Kem's Kugelrohr system. e) Schlenk flask with BTEB under inert atmosphere.

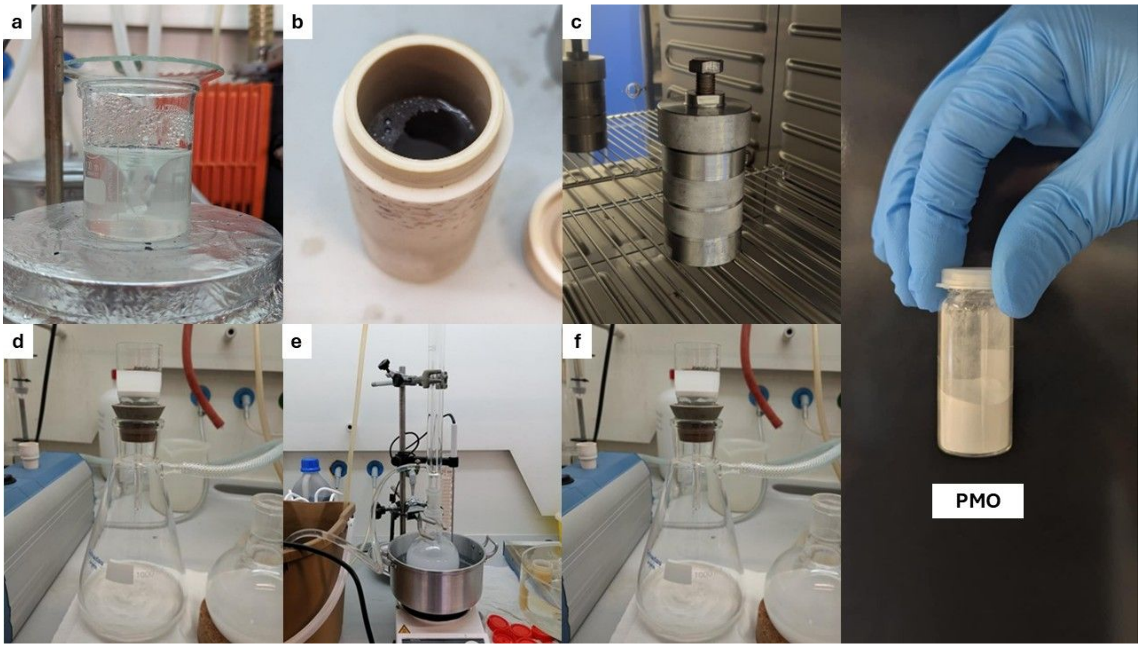

Figure S3. Step-by-step scheme for the synthesis of a Ph-PMO: a) Reaction mixture (CTAB surfactant in aqueous solution of NaOH + BTEB precursor (+ radical, for Ph-PMO_r)) stirring at room temperature for 24 h. b) Reaction mixture transferred to a teflon container before putting inside a reactor. c) Reactor in the oven for the hydrothermal treatment step (100 °C, 24 h). d) Filtration of the solid while washing with warm distilled water. e) Surfactant extraction with an acidic solution of HCl in ethanol, under reflux (80 °C) for 20 h. f) Filtration of the solid while washing with ethanol followed by distilled water.

Reproducibility

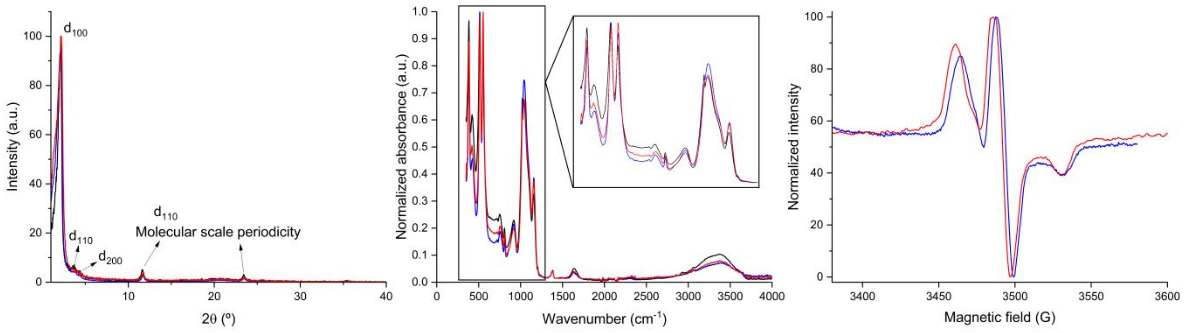

Figure S4. PXRD (left), FTIR (middle) and EPR (right) of pristine Ph-PMO (black) and two different batches of monoradical-incorporated Ph-PMO (red and blue), demonstrating reproducibility.

Table S1. Structural properties of second batch of monoradical-incorporated Ph-PMO (Ph-PMO_r2). Parameters obtained from PXRD and N_2 adsorption-desorption isotherms measured at -196 ºC.

Sample	d_{100} (nm)	a_0 (nm)	S_{BET} (m^2/g)	V_P (cm^3/g)	d_P (nm)	b (nm)
**Ph-PMO_r2**	3.99	4.61	732	0.59	2.35	2.26

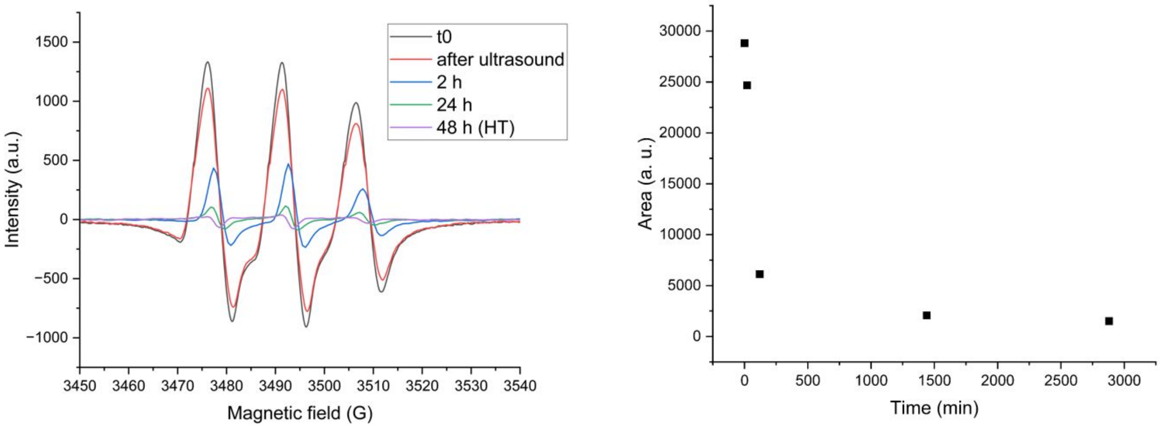

Figure S5. EPR spectra of aliquots during the 48 h reaction at different time points (left). Evolution of the integrated EPR signals as a function of time in minutes (right).

Safety measures

In addition to standard personal protective equipment (lab coat, gloves, and safety goggles), appropriate respiratory protection such as an FFP2 mask (or equivalent) should be worn when handling silica-based materials. Prolonged or repeated inhalation of respirable silica dust may result in severe respiratory diseases, including silicosis and lung cancer.
